# C57BL/6 Background Attenuates mHTT Toxicity in the Striatum of YAC128 Mice

**DOI:** 10.3390/ijms222312664

**Published:** 2021-11-23

**Authors:** Michaela K. Back, Johanna Kurzawa, Sonia Ruggieri, Jakob von Engelhardt

**Affiliations:** Institute of Pathophysiology, Focus Program Translational Neuroscience (FTN), University Medical Center of the Johannes Gutenberg University Mainz, 55128 Mainz, Germany; michaela.back@uni-mainz.de (M.K.B.); kurzawa@uni-mainz.de (J.K.); rugsonia@uni-mainz.de (S.R.)

**Keywords:** Huntington’s disease, YAC128, HdhQ150, strain background, C57BL/6, synaptic pathology, extrasynaptic NMDAR

## Abstract

Mouse models are frequently used to study Huntington’s disease (HD). The onset and severity of neuronal and behavioral pathologies vary greatly between HD mouse models, which results from different huntingtin expression levels and different CAG repeat length. HD pathology appears to depend also on the strain background of mouse models. Thus, behavioral deficits of HD mice are more severe in the FVB than in the C57BL/6 background. Alterations in medium spiny neuron (MSN) morphology and function have been well documented in young YAC128 mice in the FVB background. Here, we tested the relevance of strain background for mutant huntingtin (mHTT) toxicity on the cellular level by investigating HD pathologies in YAC128 mice in the C57BL/6 background (YAC128/BL6). Morphology, spine density, synapse function and membrane properties were not or only subtly altered in MSNs of 12-month-old YAC128/BL6 mice. Despite the mild cellular phenotype, YAC128/BL6 mice showed deficits in motor performance. More pronounced alterations in MSN function were found in the HdhQ150 mouse model in the C57BL/6 background (HdhQ150/BL6). Consistent with the differences in HD pathology, the number of inclusion bodies was considerably lower in YAC128/BL6 mice than HdhQ150/BL6 mice. This study highlights the relevance of strain background for mHTT toxicity in HD mouse models.

## 1. Introduction

Huntington’s disease (HD) is an autosomal dominant neurodegenerative disorder. HD patients show symptoms such as motor-dysfunction, starting with involuntary movement and leading to complete loss of intended motion at later disease stages; cognitive deficits; and psychiatric symptoms [1]. The cause of many of these symptoms is the neurodegeneration of the striatum, with apoptosis of GABAergic medium spiny neurons (MSNs) (reviewed in [2]). Later, during disease progression, other brain areas are also affected such as the cerebral cortex [3].

HD is a trinucleotide repeat disorder and is caused by prolonged CAG repeats (>35) in exon one of the *huntingtin* gene (*HTT*). Disease severeness correlates with the length of the trinucleotide expansion [4]. The physiological function of the huntingtin protein (HTT) includes transcriptional regulation, the transport of vesicles, and synaptic activity [5,6]. Mutant HTT (mHTT), however, disrupts these cellular processes and neuronal functions, leading to neuronal death (for review, see [7]).

HD mouse models are important tools for studying the underlying pathomechanisms and for the search for therapeutic strategies. The functional and structural analysis of brains of these mouse models as well as brains of HD patients suggests that neuronal dysfunction with alterations in synaptic function and number occurs before cell death [8,9]. There exist several HD mouse models, including transgenic and knock-in HD mouse models, which express human or mouse mHTT [10]. An extensively studied HD murine model is the YAC128 mouse [11]. This mouse model codes for the entire human *HTT* on a yeast artificial chromosome (YAC) under control of the human promoter [11]. YAC128 mice display several important pathologies that are also observed in HD patients with synapse dysfunction and spine loss in MSNs of 3-month-old mice [12], neuronal loss in the striatum at 12 months of age [11], and motor dysfunction in 4-month-old mice [13,14,15]. The striatum in YAC128 mice is the earliest but not exclusively affected brain region [16]. Due to their comparably mild and protracted phenotype, YAC128 mice are described as an ideal mouse model for studying adult-onset HD [17]. In contrast, the rapid disease progression in another mouse model, namely R6, makes it a model of juvenile HD [17]. The age of HD onset in human patients can vary from early (20–29 years), typical (30–59 years) towards late (60+ years), and disease progression and symptoms depend on age of HD onset [18]. Thus, early-onset HD patients suffer more severely from cognitive deficits than late-onset patients. In contrast, motor deficits are more pronounced in late-onset patients at already early stages of the disease [19]. Variability in onset and severity of pathology in HD mouse models can be explained by the different types of HTT with varying length of the CAG repeats and the different HTT expression levels (reviewed in [20]). For example, the truncation of mHTT in R6 mice may affect protein structure and consequently the interaction with other proteins. In addition, the truncation of mHTT affects protein localization. The truncation of mHTT therefore may increase neurotoxicity and in turn affect disease progression in HD mouse models [21]. In addition, the pathomechanisms that lead to neuron dysfunction and loss may differ for human mHTT (e.g., in YAC128, R6, BACHD) and mouse mHTT (e.g., in HdhQ150) [21]. However, strain background may contribute to variability in the onset and severity of pathology in HD mouse models, as in other neurodegenerative disease mouse models. Thus, the Aβ level in the Alzheimer’s disease mouse model 5× FAD was shown to depend on the genetic background [22]. The impact of the background strain has also been described in pain mouse models [23]. This relevance of the background strain is mainly explained by the strain-specific activity of so-called modifier-genes that influence the impact of specific gene mutations (reviewed in [24]).

YAC128 mice were generated in the FVB/N background [11], and most studies use YAC128 mice with this background [25,26,27,28,29,30,31,32]. However, the FVB/N strain is not always the optimal background. For example, FVB/N mice have retinal degeneration, which complicates many behavioral assays. Thus, YAC128 mice were used in the C57BL/6 or the 129 background by sub-breeding YAC128 mice with these strains or by sub-breeding with other transgenic or knockout mouse lines [13,33,34,35,36]. Differences in strain background may well contribute to the variability of disease onset and severity in studies using YAC128 mice. Indeed, the severity of motor dysfunction and the magnitude of the reduction in striatal volume depend on the background with the strongest pathologies in YAC128 mice with the FVB/N background and a comparably milder phenotype in mice with C57BL/6 background. Mice with the 129 background showed intermediate phenotype severity [13]. Similarly, the dependency of the onset and severity of behavioral deficits on the strain background was demonstrated in another study that investigated different HD mouse models, including R6/2, HdhQ111, BACHD and YAC128 mice [37]. Importantly, behavior such as motor performance on the Rotarod, activity and rearing in the Open Field test, anxiety, and startle response is very variable in wildtype mice with different backgrounds (FVB/N and C57BL/6) [37]. Thus, the interpretation that the variability of behavioral deficits in YAC128 mice results from strain-specific differences in mHTT toxicity is somewhat confounded by strain-specific differences in behavior.

We therefore wondered whether the comparably mild behavioral deficits in YAC128 mice in C57BL/6 background are reflected by resistance of MSNs to mHTT toxicity. YAC128 mice have frequently been used to investigate early pathologies of HD such as spine loss, synaptic dysfunction and alterations in passive electrophysiological properties. These pathologies have been well documented in 3–12-month-old YAC128 mice with FVB/N background [12,38].

In this study, we investigated spine density and dendritic arborization, synaptic function and passive electrophysiological properties of MSN from YAC128 mice in the C57BL/6N background. We observed comparably mild alterations in YAC128/BL6 mice even at an age of one year. As described previously, motor behavior was altered in YAC128/BL6 mice. In order to study whether the C57BL/6 strain background per se averts mHTT toxicity, we used the HdhQ150 mouse model, which has been generated in a mixed 129/Ola and C57BL/6 background [39] and is frequently used in a 75–90% C57BL/6 background [10]. Synapse dysfunction was observed in 6-month-old HhdQ150/BL6 mice. Consistent with the difference in mHTT toxicity, we observed a considerably higher number of inclusion bodies in the striatum of HhdQ150/BL6 than in the striatum of YAC128/BL6.

In summary, our results suggest that strain background in combination with the propensity to form inclusion bodies influences the severity and onset of disease pathology in the striatum of HD mouse models.

## 2. Results

### 2.1. Normal Neuron Morphology and Spine Number in 12-Month-Old YAC128/BL6 Mice

**Figure 1:**
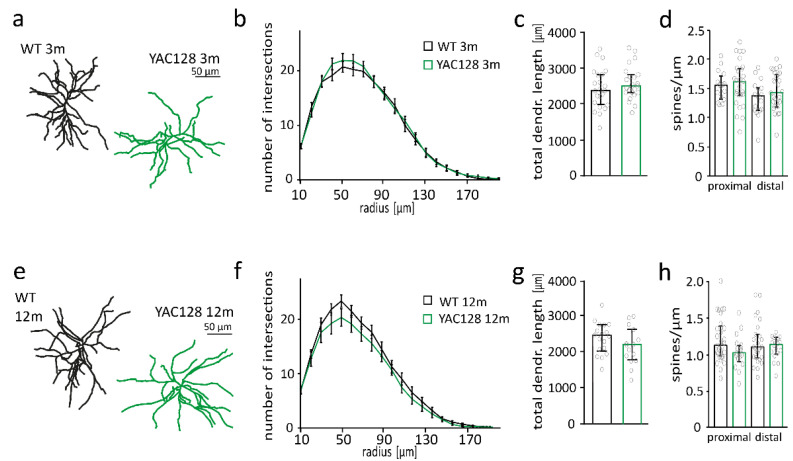
Neuron morphology is unaltered in 3- and 12-month-old YAC128/BL6 mice. (**a**) Examples of reconstructed medium spiny neurons (MSNs) from 3-month-old WT and YAC128/BL6 mice. (**b**) Sholl analysis with number of dendrite intersections and (**c**) bar graph of total dendritic length of MSNs from WT (*n* = 23) and YAC128/BL6 (*n* = 23) mice. (**d**) Bar graph of spine number per micrometer of proximal and distal dendrites of MSNs from WT (proximal *n* = 16, distal *n* = 17) and YAC128/BL6 (proximal *n* = 27, distal *n* = 29) mice. (**e**) Examples of reconstructed of MSNs from 12-month-old WT and YAC128/BL6 mice. (**f**) Sholl analysis with number of dendrite intersections and (**g**) bar graph of total dendritic length of MSNs from WT (*n* = 18) and YAC128/BL6 (*n* = 15) mice. (**h**) Bar graph of spine number per micrometer of proximal and distal dendrites of MSNs from WT (proximal *n* = 29, distal *n* = 27) and YAC128/BL6 (proximal *n* = 27, distal *n* = 29) mice. Bar graphs are depicted as median ± IQR.

Morphological changes in MSNs precede neuron loss and are an early indicator of HD pathology [40]. Spine density is mildly reduced in 3–4-month-old YAC128 and strongly reduced by more than 30% in YAC128/FVB mice at an age of 12 months [12,31,41,42]. To investigate the relevance of the strain background on mHTT toxicity, we quantified spine number in YAC128 mice in the C57BL/6 background (YAC128/BL6). The number of spines in proximal, and distal dendrites of MSNs were not different between 3-month-old YAC128/BL6 mice and control littermates (proximal: *t*-test; *p* = 0.6939; distal: *t*-test; *p* = 0.1947) (Figure 1d). Spine number was also not reduced in MSNs of 12-month-old YAC128/BL6 mice (*t*-test; *p* = 0.0697) (Figure 1h). Sholl analysis of biocytin-filled and reconstructed MSNs revealed no difference in dendritic arborization as well as total dendritic length between 3-month or 12-month-old WT and YAC128/BL6 mice (*t*-test; 3 months: *p* = 0.311; 12 months: *p* = 0.1775) (Figure 1a–c,e–g). Thus, we observed no changes in MSN morphology in both 3-month and 12-month-old YAC128/BL6 mice in contrast to the alterations in MSN morphology with reduced spine density in 3- and 12-month-old YAC128/FVB mice [12,41,42].

### 2.2. Subtle Changes in Synapse Function in 12-Month-Old YAC128/BL6 Mice

**Figure 2:**
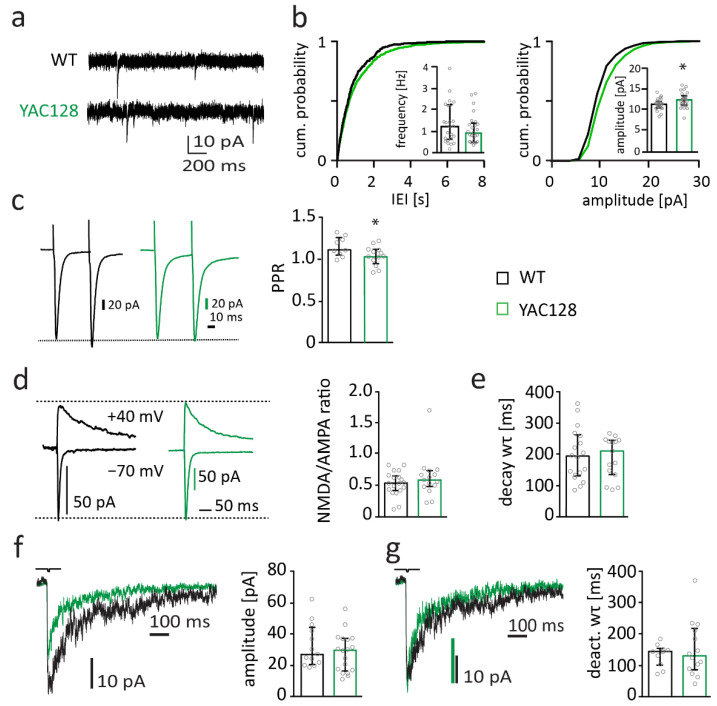
Synapse function is subtly changed in 12-month-old YAC128/BL6 mice. (**a**) Sample traces of miniature excitatory postsynaptic currents (mEPSC) recordings from MSNs of WT and YAC128/BL6 mice. (**b**) Cumulative distribution plot of AMPAR-mediated miniature excitatory postsynaptic currents (mEPSC) inter-event-intervals (IEIs) and amplitude. The inserts show bar graphs of the median mEPSC frequency and amplitude of MSNs from WT (black, *n* = 24) and YAC128/BL6 (green, *n* = 26) mice. (**c**) Sample traces of paired-pulse (PP) recordings from MSNs of WT (black) and YAC128/BL6 mice (green). Currents were evoked with an inter-pulse-interval of 50 ms. Bar graph of PP ratio (PPR) in MSNs of WT (*n* = 9) and YAC128/BL6 (*n* = 13) mice. (**d**) Sample traces of NMDAR- and AMPAR-mediated currents recorded at a holding potential of +40 mV and −70 mV, respectively. Bar graph of NMDA/AMPA ratio of MSNs from WT (*n* = 19) and YAC128/BL6 (*n* = 15) mice. (**e**) Bar graph of the weighted time constant (wτ) of synaptic NMDAR-mediated current decays of MSNs from WT (*n* = 18) and YAC128/BL6 (*n* = 15) mice. (**f**) Sample traces of extrasynaptic NMDAR-mediated currents recorded from nucleated patches of MSNs and bar graph of median peak current amplitudes of MSNs from WT (*n* = 13) and YAC128/BL6 (*n* = 18) mice. (**g**) Peak-normalized sample traces of extrasynaptic NMDAR-mediated currents recorded from nucleated patches of MSNs and bar graph of the weighted time constant (wτ) of current deactivation in nucleated patches of MSNs of WT (*n* = 10) and YAC128/BL6 (*n* = 14) mice. Bar graphs are depicted as median ± IQR. * = *p* < 0.05.

Synaptic dysfunction of MSNs occurs early in HD patients and in HD mouse models [8,43,44]. Thus, alterations in synaptic function are present in YAC128 mice with FVB background already at an age of 1.5 months [45,46,47]. At the age of 12 months and older, synaptic function of MSNs from YAC128/FVB mice is severely disturbed [45,46,48]. To investigate whether the strain background affects the occurrence of synaptic dysfunction in YAC128 mice, we recorded α-amino-3-hydroxy-5-methyl-4-isoxazolepropionic acid receptor (AMPAR)-mediated miniature excitatory postsynaptic currents (mEPSCs) in striatal MSNs of YAC128/BL6 and WT mice. Neither mEPSC frequency nor amplitude were different between genotypes in 3-month-old mice (Supplementary Figure S1b). mEPSC frequency was also not affected in 12-month-old YAC128/BL6 mice (there was a trend to reduced frequency in YAC128/BL6 mice; Mann–Whitney test, *p* = 0.435) (Figure 2a,b). There was a small but significant increase in mEPSC peak amplitude in MSNs from YAC128/BL6 mice (*t*-test, *p* = 0.021) (Figure 2a,b). Considering the trend to reduced mEPSC frequency in YAC128/BL6 mice, we wondered whether there is a significant reduction in older YAC128/BL6 mice. However, neither mEPSC frequency nor amplitude were altered in 18-month-old YAC128/BL6 mice when compared to WT littermates (Supplementary Figure S1d).

Presynaptic function with altered vesicle release probability has been observed in YAC128/FVB mice. Thus, the paired-pulse ratio (PPR), which changes with alterations in presynaptic vesicle release probability, is decreased in 1.5-month–old mice and increased in 12-month-old YAC128/FVB mice [45]. To investigate presynaptic function, we investigated PPR of evoked EPSCs with an inter-stimulus-interval of 50 ms. PPR was slightly reduced in MSNs of 12-month-old YAC128/BL6 mice compared to their littermate controls (*t*-test; *p* = 0.0426) (Figure 2c). The decrease in PPR suggests that vesicle release probability is reduced in YAC128/BL6 mice and may explain the trend to reduced mEPSC frequency. Interestingly, the reduced PPR in 12-month-old YAC128/BL6 mice resembles the PPR change in 1.5-month-old YAC128/FVB mice and is opposite to the increase in PPR in 12-month-old YAC128/FVB mice [45].

N-methyl-D-aspartate receptor (NMDAR) expression increases, and its function changes, in HD mouse models [49]. Expression of NMDARs increases in the striatum of YAC128 mice already at an age of one month prior to occurrence of neuropathology and behavioral deficits [50,51]. These alterations in NMDAR expression and function are considered to play an important role in HD pathophysiology [52,53]. Synaptic NMDAR currents are increased in 1-month and 12-month-old YAC128 mice with FVB background [51]. To assess synaptic NMDAR function, we recorded evoked NMDAR-mediated currents in MSNs. The amplitude of NMDAR-mediated currents was normalized to the amplitude of AMPAR-mediated currents. The NMDA/AMPA-ratio was unaltered in YAC128/BL6 mice (Mann–Whitney test; *p* = 0.4102) (Figure 2d), suggesting that the number of synaptic NMDARs relative to the number of synaptic AMPARs is not altered in MSNs of 12-month-old YAC128/BL6 mice. Considering that AMPAR-mediated EPSC amplitudes were only slightly increased (Figure 2a), this indicates that there may be a small increase in the number of synaptic NMDARs. NMDARs of striatal MSNs are known to contain mainly GluN2A and GluN2B subunits [54,55]. The composition of NMDARs determines current kinetics. GluN2B-containing NMDARs deactivate considerably more slowly than GluN2A-containing NMDARs [56]. Similar decay kinetics of synaptic NMDAR-mediated currents in MSNs of YAC128/BL6 and WT mice (Figure 2e, *t*-test; *p* = 0.7485) suggest that NMDAR subunit composition is not different between genotypes.

The expression of extrasynaptic GluN2B-containing NMDARs increases in MSNs of 1-month-old YAC128 with FVB background [50,51]. This increase is thought to be relevant for HD pathophysiology [53] as the activation of extrasynaptic GluN2B-containing NMDARs induces cell-death pathways [57,58]. We activated extrasynaptic NMDARs by ultra-fast application of glutamate onto nucleated patches of MSNs. Amplitude and deactivation kinetics of extrasynaptic NMDAR-mediated currents were not affected in in MSNs of 12-month-old YAC128/BL6 mice (*t*-test; *p* = 0.349 and *p* = 0.5273, respectively) (Figure 2f,g), indicating that number and composition of extrasynaptic NMDARs are not different between genotypes.

Thus, the electrophysiological analyses revealed only minor alterations in synaptic function and no change in NMDAR-mediated currents in MSNs of 12-month-old YAC128/BL6 mice.

### 2.3. Subtle Changes in Active Membrane Properties of MSNs in 12-Month-Old YAC128/BL6 Mice

**Figure 3:**
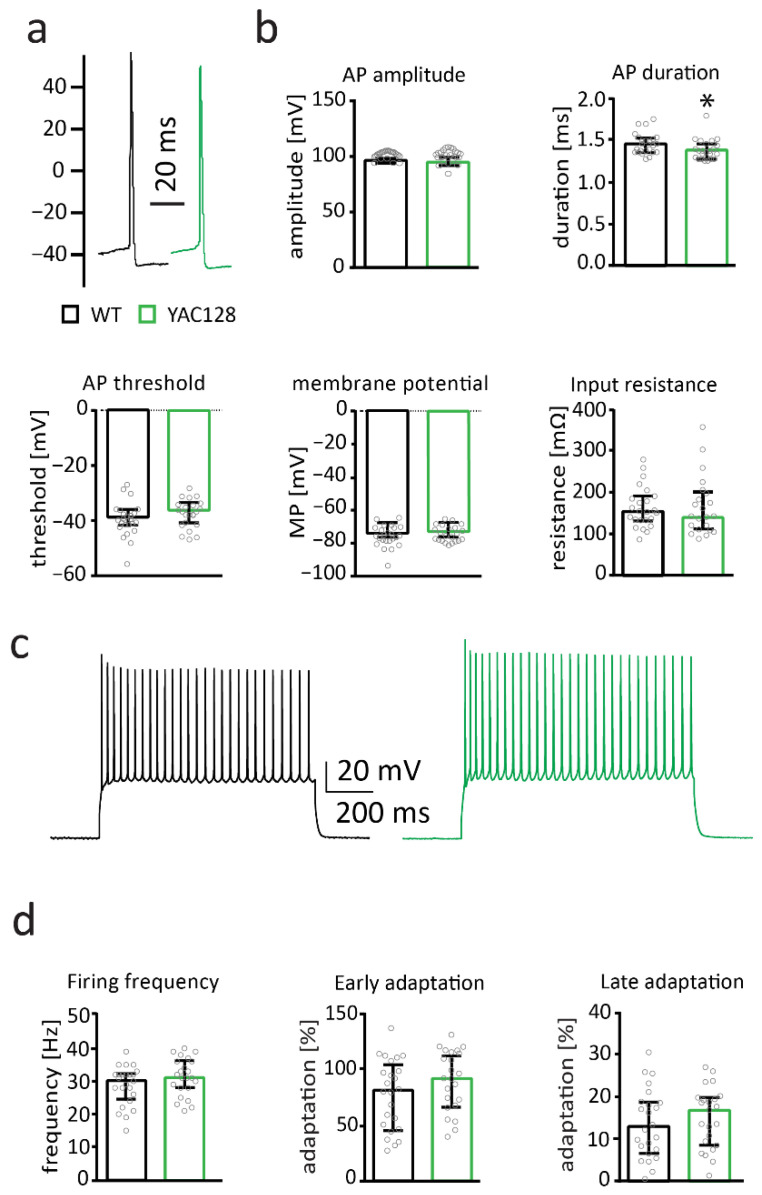
Membrane properties are by and large unaltered in 12-month-old YAC128/BL6 mice. (**a**) Sample traces of action potentials (APs) recorded from MSNs of WT (black) and YAC128/BL6 (green) mice. (**b**) Bar graphs of AP threshold, amplitude, threshold and input resistance (WT *n* = 24; YAC128/BL6 *n* = 23). (**c**) Sample traces of AP firing induced by current injection. (**d**) Bar graphs of firing frequency, and early and late adaptation of MSNs from WT (*n* = 24) and YAC128 (*n* = 23) mice. MP = membrane potential. Bar graphs are depicted as median ± IQR. * = *p* < 0.05.

Electrophysiological membrane properties of MSNs are affected in 7- to 12-month-old YAC128 mice in the FVB background [38,50]. We studied basic membrane properties in MSNs from 12-month-old YAC128/BL6 mice and littermate controls. There was no genotype difference in input resistance (Mann–Whitney test; *p* = 0.748) and membrane potential (*t*-test; *p* = 0.4122) (Figure 3b). Additionally, action potential threshold (*t*-test; *p* = 0.2182) and amplitude (*t*-test; *p* = 0.4666) were not affected in MSNs from 12-month-old YAC128/BL6 mice (Figure 3b). Action potential duration was slightly decreased in MSNs from YAC128/BL6 mice (Mann–Whitney test; *p* = 0.0297) (Figure 3b).

Firing properties of YAC128/BL6 MSNs were analyzed by maximal current injections. Maximal firing frequency (*t*-test; *p* = 0.1392), early spike adaptation (*t*-test; *p* = 0.1415) and late spike adaptation (*t*-test; *p* = 0.539) were not altered in MSNs from 12-month-old YAC128/BL6 mice (Figure 3d). This indicates that passive and active membrane properties of MSNs are only mildly affected in 12-month-old YAC128/BL6 mice.

### 2.4. Early Synaptic Deficits in MSNs of HdhQ150/BL6 Mice

**Figure 4:**
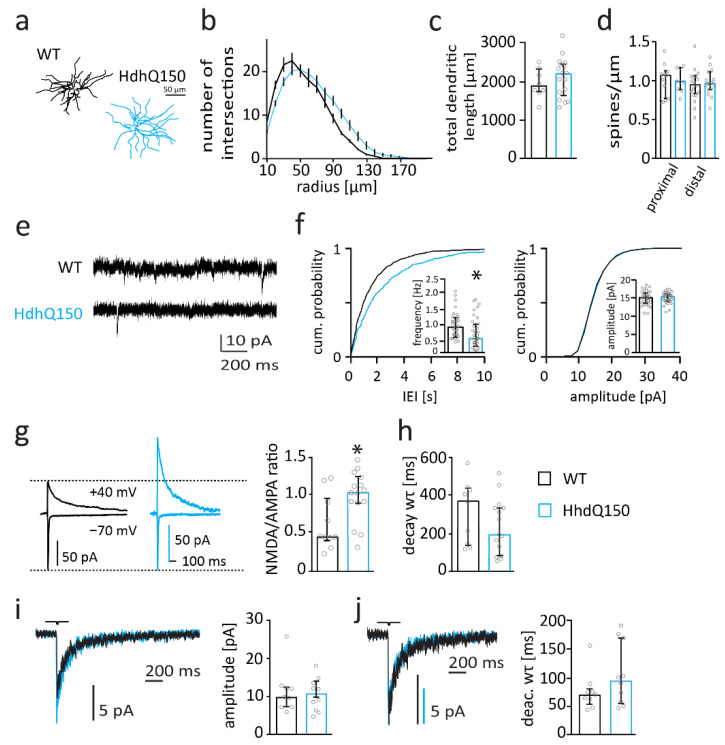
Synaptic function is impaired in 6-month-old HdhQ150/BL6 mice. (**a**) Example reconstructions of MSNS. (**b**) Sholl analysis with number of dendrite intersections per radius and (**c**) bar graph of total dendritic length of MSNs from WT (*n* = 8) and HdhQ150/BL6 (*n* = 17) mice. (**d**) Bar graph of spine number per µm of MSNs from WT (*n* = 10) and HdhQ150/BL6 (*n* = 18) mice. (**e**) Sample traces of mEPSC recordings from MSNs from 6-month-old WT and HdhQ150/BL6 mice. (**f**) Cumulative distribution plot of mEPSC IEIs and amplitude as well as bar graphs of the median mEPSC frequency and amplitude of MSNs from 6-month-old WT (black, *n* = 31) and HdhQ150/BL6 (blue, *n* = 32) mice. (**g**) Sample traces of NMDAR- and AMPAR-mediated currents recorded at a holding potential of +40 mV and −70 mV, respectively, and bar graph of the NMDA/AMPA-ratio of MSNs from WT (*n* = 10) and HdhQ150/BL6 (*n* = 15) mice. (**h**) Bar graph of the time constant (wτ) of NDMAR-mediated current decay in MSNs of WT (*n* = 9) and HdhQ150/BL6 (*n* = 15) mice. (**i**) Sample traces of extrasynaptic NMDAR-mediated currents recorded in nucleated patches and bar graph of peak current amplitudes of MSNs from WT (*n* = 9) and HdhQ150/BL6 (*n* = 10) mice. (**j**) Peak-scaled sample traces of extrasynaptic NMDAR-mediated currents recorded from nucleated patches, and bar graph of the deactivation time constant (wτ) of currents from MSNs of WT (*n* = 9) and HdhQ150/BL6 (*n* = 10) mice. Bar graphs are depicted as median ± IQR. * = *p* < 0.05.

The analyses with YAC128 mice showed that alterations of MSN morphology and function are very mild in the C56BL/6 background. We wondered whether that holds true also for mice with higher mHTT expression. The HdhQ150 mouse model is a well-studied HD model that is bred in the C57BL/6 background but nevertheless shows early behavioral deficits [59]. Morphological analysis revealed that overall dendritic length of MSNs was not altered in 6-month-old HdhQ150/BL6 mice compared to WT littermates (Figure 4c). However, Sholl analysis revealed subtle changes in neuronal arborization with an increase in the number of intersections per radius at distal dendritic compartments of MSNs in HdhQ150/BL6 mice (2-way-ANOVA with Sidak’s multiple comparison test) (Figure 4b). Spine number was not affected in proximal (*t*-test; *p* = 0.5096) and distal (*t*-test; *p* = 0.6851) dendrite compartments of MSNs in HdhQ150/BL6 mice (Figure 4d).

Electrophysiological analysis revealed a reduced mEPSC frequency (*t*-test; *p* = 0.0331) and a corresponding right-shift of the cumulative distribution curve for mEPSC inter-event intervals in MSNs from HdhQ150/BL6 mice (Figure 4e,f). mEPSC amplitudes were not different between genotypes (*t*-test; *p* = 0.7829) (Figure 4f). The reduction in mEPSC frequency suggests that functional synapse number is reduced in HdhQ150/BL6 mice, which is consistent with previous observations in R6/2 and Q175 mice [60,61].

Importantly, the NMDAR/AMPAR-ratio was increased in MSNs of 6-month-old HdhQ150/BL6 mice compared to that in WT mice (*t*-test; *p* = 0.0187) (Figure 4g). Considering that the amplitude of AMPAR-mediated mEPSCs did not change, these data suggest that the expression of synaptic NMDARs is increased in MSNs of HdhQ150/BL6 mice.

The decay of NMDAR-mediated currents was not affected (*t*-test; *p* = 0.2411) (Figure 4h), indicating that the composition of synaptic NMDARs is not strongly altered in MSNs of HdhQ150/BL6 mice. There was, however, a trend to a reduced decay time constant, which may indicate an increased contribution of GluN2A-containing NMDARs. Extrasynaptic NMDAR-mediated currents were not affected in MSNs of HdhQ150/BL6 mice (Figure 4i). Thus, peak amplitude (Mann–Whitney test; *p* = 0.9205) and deactivation time constant of NMDAR-mediated currents (Mann–Whitney test; *p* = 0.211) were similar between genotypes (Figure 4i,j).

In summary, we found synaptic and morphological changes in MSNs of 6-month-old HdhQ150 mice. Thus, MSNs of HdhQ150/BL6 mice display more severe and earlier alterations in both MSN morphology and synapse function than MSNs of YAC128/BL6 mice. Moreover, this suggests that the C57BL/6 background provides only partial protection against mHTT toxicity (or protection only in the YAC128 mouse model).

### 2.5. Smaller Number of Inclusion Bodies in YAC128/BL6 Mice Than in HhdQ150/BL6 Mice

Considering the mild anatomical and functional phenotype in 12-month-old YAC128/BL6 mice, we wondered whether human mHTT is expressed and aggregates in inclusion bodies, which are a hallmark of HD [62]. Interestingly, it was previously shown that the expression level of mHTT is similar in YAC128 mice with different background strains (FVB, C57BL/6 and 129) [13], suggesting that the strain-specific variability in mHTT toxicity does not result from differences in mHTT expression. However, the same study showed that the occurrence of nuclear localization of mHTT, an early hallmark of HD, is different in the three backgrounds strains studied [13]. Thus, nuclear accumulation of mHTT was already found in 1-month-old YAC128/FVB mice but only from 4 months onwards in YAC128/BL6 mice [13].

**Figure 5:**
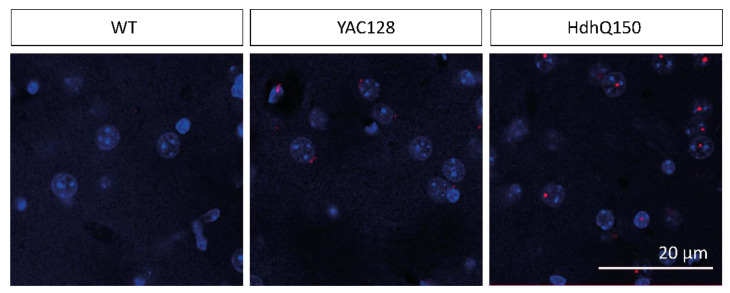
The number of inclusion bodies is lower in YAC128/BL6 than in HdhQ150Q/BL6 mice. Confocal microscopy images of brain slices of the striatum of WT (12 month), YAC128/BL6 (12 month) and HdhQ150 (10 month) mice. Inclusion bodies (red) were visualized with an MW8 antibody and cell nuclei with DAPI staining (blue). Merged images show the inclusion bodies with an intranuclear localization in HdhQ150 animals.

We detected few inclusion bodies in the striatum of 12-month-old YAC128/BL6 mice as investigated by immunohistochemistry using an antibody specific for human mHTT (Figure 5). No inclusion bodies were observed in C57BL/6 control mice (WT) (Figure 5). Importantly, the number of inclusion bodies was considerably higher in the brain of HdhQ150/BL6 mice than in the brain of YAC128/BL6 mice (Figure 5).

### 2.6. Impaired Behavior in YAC128/BL6 Mice

Considering the low number of inclusion bodies and the subtle alterations in neuron morphology, synaptic function and membrane properties, we wondered whether behavior is affected in YAC128/BL6 mice.

**Figure 6:**
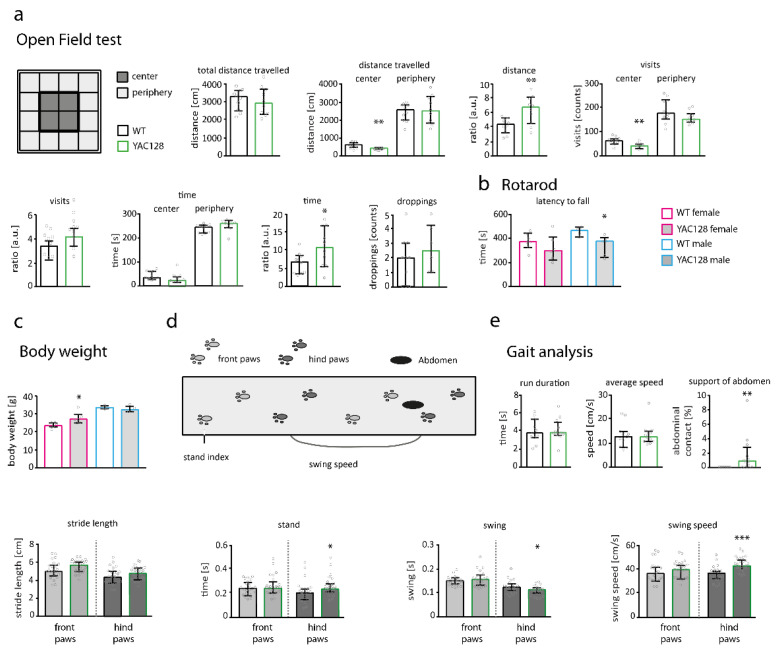
Behavior is impaired in 9-month-old YAC128/BL6 mice. (**a**—**left**) Scheme of the Open Field divisions with center and periphery. (**a**—**right**) Bar graphs of Open Field behavioral parameters of WT (*n* = 10) and YAC128/BL6 (*n* = 10) mice. (**b**) Bar graph of the latency to fall off the Rotarod for male and female WT (n females = 7; males = 6) and YAC128/BL6 (n females = 4, males = 5) mice. (**c**) Bar graph of the median body weight of female and male of WT and YAC128/BL6 mice. (**d**) Scheme of the gait analysis (Catwalk) setup. (**e**) Bar graphs of gait analysis parameters from WT (*n* = 11) and YAC128/BL6 (*n* = 12) mice. Bar graphs are depicted as median ± IQR. * = *p* < 0.05, ** = *p* < 0.01, *** = *p* < 0.001.

General activity and exploratory behavior were assessed using the Open Field test. No difference was observed in motor activity between 9-month-old YAC128/BL6 and control mice, as quantified by the total distance travelled (*t*-test; *p* = 0.7556) (Figure 6a). Mice of both genotypes did also not differ in the distance travelled (*t*-test; *p* = 0.7131), time spent (Mann–Whitney test; *p* = 0.0524) and number of visits (*t*-test; *p* = 0.1162) in the periphery of the Open Field (Figure 6a). However, YAC128/BL6 travelled significantly less distance in the center of the Open Field than control mice (*t*-test; *p* = 0.004; Figure 6a). Consequently, the ratio of travelled distance periphery/center was also significantly different between YAC128/BL6 and control mice (*t*-test; *p* = 0.0075; Figure 6a). Despite a lower number of visits of YAC128/BL6 mice in the center (*t*-test; *p* = 0.0061), the ratio of visits in periphery/center was not significantly different (*t*-test; *p* = 0.0551) (Figure 6a). Finally, although YAC128/BL6 mice spent a comparable amount of time in the center of the Open Field (Mann–Whitney test; *p* = 0.0753), the ratio of time spent in periphery/center was significantly increased (*t*-test; *p* = 0.034; Figure 6a). Findings of the Open Field test suggest that overall motor activity is unaltered but anxiety levels increased in YAC128/BL6 mice. Interestingly, when data was separated by sex, both female and male showed deficits in the Open Field (Supplementary Figure S2a). Female mice travelled less distance in the center of the Open Field (*t*-test; *p* = 0.0093), while male mice visited the center less often (*t*-test; *p* = 0.0267) (Supplementary Figure S2a).

Motor performance in 9-month-old YAC128/BL6 mice was additionally studied using the Rotarod test. Since body weight can influence the fall latency in Rotarod experiments, we measured the weight of the animals. Male YAC128/BL6 mice had a similar weight compared to male WT mice (Figure 6c), and stayed for significantly less time on the Rotarod (*t*-test; *p* = 0.0364; Figure 6b) than male control mice. Although female YAC128/BL6 mice were significantly heavier than female WT (*t*-test; *p* = 0.01) (Figure 6c), fall latency of female mice was not different between genotypes (*t*-test; *p* = 0.2873) (Figure 6b).

Finally, we employed the Catwalk to detect more subtle alterations in motor performance. Average speed and run duration were similar in WT and YAC128/BL6 mice (Figure 6e). YAC128/BL6 mice also showed no deficits in stride length (Figure 6e). We observed an increased swing speed in hind paws of YAC128/BL6 mice (*t*-test; *p* < 0.0001). Consistently, the swing phase time, which describes the time that the paw is in the air, was decreased in hind paws from YAC128/BL6 mice (swing; Mann–Whitney test: *p* = 0.0311) (Figure 6e). Stand (time of paw contact with the glass plate) was increased in hind paws of YAC128/BL6 mice (quantified for front and hind paws separately; Mann–Whitney test: *p* = 0.0348) (Figure 6e). We also observed an increased abdominal contact with the glass plate in YAC128/BL6 mice compared to WT mice (Mann–Whitney test; *p* = 0.0024). The increased stand and swing speed of the hind paws, together with the abdominal support, indicate the need of YAC128/BL6 mice for more support during body movement (Figure 6e). Deficits in motor performance observed in the Catwalk were also apparent when data was analyzed for female and male mice separately (Supplementary Figure S2e)

In summary, behavior analysis of YAC128 mice in the C57BL/6 background revealed motor impairments, consistent with a dysfunction in striatal signaling.

## 3. Discussion

Our data suggest that the strain background affects the phenotype of YAC128 mice. Analysis of MSNs revealed that YAC128 mice in the C57BL/6 background are only mildly affected. This is especially evident for early HD-related perturbations at the synaptic level and less evident for the behavioral phenotype.

HD pathology in humans and mouse models starts with neuronal dysfunction before the onset of cognitive and motor symptoms [9,53,63,64,65,66,67,68,69,70]. The underlying mechanisms leading to neuronal and synaptic dysfunction have, however, not yet been fully elucidated. Altered NMDAR function and expression in striatal MSNs is thought to be involved [49,52,71,72,73,74,75,76]. It was previously shown that NMDAR-mediated currents increase in MSNs of HD mouse models, which could lead to excitotoxicity (reviewed in [52,77]). Specifically, an upregulation of the expression of extrasynaptic NMDARs was observed, which is supposed to trigger cell death signaling [51,78,79,80]. Post-mortem analysis of the brains of HD patients revealed an upregulation of NMDAR mRNA expression at early stages of the disease [81]. In contrast, NMDARs decrease at later disease stages, which may be explained by the loss of neurons [81,82]. Early disturbances at synaptic and extrasynaptic sites may cause neurodegeneration in the striatum and consequently behavioral deficits [83]. It is therefore of importance to recapitulate early synaptic perturbations in HD mouse models in order to investigate their involvement in HD pathophysiology and to consider possible early therapies for HD.

The first HD mouse model (R6/2) was developed in 1996 [84]. R6/2 mice, which express a truncated mHTT, are still widely used for studying the pathomechanisms of HD [37,38,84,85]. R6/2 mice die around the age of 3–4 months and may thereby reflect more the pathogenesis in juvenile and early-onset HD patients. Two other widely used HD mouse models are YAC128 (expression of full-length human mHTT via a yeast artificial chromosome; [11]) and HdhQ150 (targeted knock-in of chimeric mouse/human mHTT exon1 containing 150 CAG repeats; [39]). These mouse models vary in the way they were generated, how they drive the expression of the mutated HTT, whether they express a truncated or full-length HTT, CAG repeat length, and strain background. Consequently, HD symptoms and their progression are different in these mouse models.

Laboratory mice are available in different strain backgrounds. Each strain shows distinct genotypic and phenotypic properties. However, differences within the genome of these strains can influence the phenotype (reviewed in [86]). This can be explained by the expression of modifier-genes that influence the impact of specific gene mutations (reviewed in [24]). The genetic background was already shown to influence the severity of other neurodegenerative diseases [87,88,89,90]. As outlined above, variability in occurrence and severity of HD symptoms in HD mouse models can be explained by different lengths of CAG repeats or the type of transgenic model used. However, it is very likely that the genetic background also influences HD pathology in model mice.

YAC128 mice were originally generated and described in the FVB background [11]. This strain background is known for its high susceptibility to excitotoxicity [91] and thus is relevant to neurodegeneration. However, the C57BL/6 background is the more widely used background in transgenic mouse models. Mouse lines are often kept in the C57BL/6 (or a mixed) background when sub-breeding two mouse lines. Regarding neurotoxicity, striatal neurons of C57BL/6 mice are, in contrast to those in FVB mice, resistant to quinoline-induced neurodegeneration [92]. We thus wondered whether YAC128 mice in the C57BL/6 background were less susceptible to early synaptic perturbation than YAC128 mice in the FVB background.

Only a few studies have so far investigated the YAC128 mouse model in C57BL/6 background [13,33,34,35,36,93]. A detailed comparison of the behavioral phenotype of the YAC128 line in the C57BL/6 and FVB background was performed by van Raamsdonk and colleagues [13]. At an age of 12 months, YAC128/FVB mice showed a more severe behavioral phenotype than YAC128/BL6 mice. For example, motor performance on the Rotarod was more impaired and hypoactivity in the Open Field more pronounced in YAC128/FVB then in YAC128/BL6 mice [13]. Deficits of performance in motor and learning tasks are already present in younger YAC128/BL6 mice. For example, performance on the Rotarod is already disturbed at an age of 4 months [34]. Similar to Raamsdonk and colleagues, we observed only mild behavioral deficits in the Open Field and Rotarod in 12-month-old YAC128/BL6 mice. Additionally, we identified subtle changes in the gait of YAC128/BL6 mice. HD patients also suffer from gait abnormalities such as spontaneous knee flexion; changed swing parameters; and decreased step frequency, stride length and velocity [94]. Thus, despite their weaker phenotype and later onset of symptoms, YAC128/BL6 mice might be useful for studying early signs of motor deficits in HD. This is of interest as some behavioral experiments cannot be performed with YAC128/FVB background. For example, FVB mice suffer from retinal degeneration, which excludes all behavior experiments involving visual stimuli (https://www.jax.org/strain/027432, accessed on 21 October 2021).

Consistent with the subtle changes in behavior in 12-month-old YAC128/BL6 mice, MSN morphology, spine density, functional synapse number, synapse function, synaptic and extrasynaptic NMDAR-mediated currents, and membrane properties were, by and large, unaltered. Similar analyses revealed significant alterations in these parameters in YAC128/FVB mice already at a much younger age. For example, the amplitude of extrasynaptic NMDAR-mediated currents increases already in 1-month-old YAC128/FVB mice [51]. In addition, spine density is already reduced in 4-month-old YAC128/FVB mice [42]. In contrast, spine density and amplitude of extrasynaptic NMDAR-mediated currents were not affected in 12-month-old YAC128/BL6 mice. The absence of strong alterations in MSN function is consistent with the low number of inclusion bodies in the striatum of 12-month-old YAC128/BL6 mice. Interestingly, it has previously been shown that the mHTT levels is comparable in YAC128 mice in different background strains (FVB, C57BL/6 and 129) [13]. However, the amount of nuclear localization of mHTT is considerably lower in YAC128/BL6 mice than in YAC128/FVB or YAC128/129 mice [13]. Thus, the strain-specific differences in severity of neuropathological alterations do not result from differences in the expression of mHTT but rather from differences in the amount of nuclear localization of mHTT. Nuclear and cytoplasmic inclusion bodies are found in the brain of HD patients [95]. Interestingly, the number of nuclear inclusion bodies correlates with disease severity and is considerably higher in the brain of early-onset than in the brain of adult-onset HD patients [96]. In contrast, cytoplasmic inclusion bodies are more prevalent in adult-onset than in juvenile-onset HD [95]. Thus, the nuclear localization of inclusion bodies in HdhQ150/BL6 resembles that in juvenile-onset HD patients, whereas the cytoplasmic localization of inclusion bodes might indicate that pathomechanisms in YAC128/BL6 mice are more similar to that in adult or late-onset HD patients.

HdhQ150/BL6 mice display HD-specific neuropathological alterations already at an age of 6 months. The more severe phenotype of these mice compared to that of YAC128/BL6 mice is consistent with the higher number of inclusion bodies. The alterations in MSN function in HdhQ150/BL6 mice show that mHTT-mediated toxicity occurs also in the C57BL/6 background. That suggests that mHTT is also toxic in the C57BL/6 background if mHTT is translocated into the nucleus [13].

## 4. Conclusions

Our data highlight the limitations of the YAC128 mouse model in the C57BL/6 background for the investigation of cellular HD pathologies. Thus, it appears to be favorable to use YAC128 mice in the FVB background when studying HD-mediated cellular pathologies. In addition, sufficient numbers of backcrossings are essential when crossing YAC128/FVB mice with transgenic or knockout mice in C57BL/6 background. However, YAC128 mice in the C57BL/6 background might be useful when investigating HD pathology with behavioral tests that require normal vision or when investigating pathomechanisms of adult- or late-onset HD. In this case, the mice should be sufficiently old.

We also conclude from our study that it is not the C57BL/6 background alone that explains the mild HD pathology since HdhQ150 mice in the C57BL/6 background show more severe HD-related deficits on the synaptic and behavior level. Thus, we suggest that it is the combination of a specific HD mouse model and the strain background that defines severity and onset of HD symptoms.

Finally, the strain-specific differences in mHTT toxicity may be important since a comparison of gene expression in the different mouse strains could help to identify modifier genes. The discovery of HD modifier genes might help to better understand the regulation of disease onset and progression. This information might also be of value for achieving a better understanding of the relevance of strain background in mouse models of other neurodegenerative diseases.

## 5. Materials and Methods

### 5.1. Mouse Lines

All animal experiments as well as breedings were carried out in compliance with the German Welfare Act and approved by the Governmental Supervisory Panel on Animal Experiments of Baden-Wuerttemberg (G-102/16), Karlsruhe and Rhineland Palatinate, Koblenz (G-16-1-069). All procedures followed the “Principles of laboratory animal care” (NIH publication No. 86–23, revised 1985).

Mice had access to food and water ad libitum and were housed in groups of up to four animals in polycarbonate boxes in an inverted 12 h light/12 h dark cycle. Cages were changed at least once per week

YAC128 [11]; B6.FVBTg(YAC128)53Hay/ChdiJ; Jackson lab strain 027432) and Hdh (CAG)150 mice, named HdhQ150 in this study (B6.129P2-Htttm2Detl/150J; [39]), were used as mouse models for HD. The YAC128 line was further sub-bred with C57BL/6N mice. YAC128 mice carry a yeast artificial chromosome coding for the human HTT with 128 CAG triplet repeats. The HdhQ150 knock-in mouse model expresses the endogenous mouse HTT with 150 CAG repeats. This mouse model was originally created in a mixed 129/Ola and C57BL/6J background [39] and is now used in 75–90% C57BL/6 background [10].

Mice of both sexes were used for experiments. Non-transgenic littermates were used as controls in all experimental conditions.

### 5.2. Electrophysiology

YAC128/BL6 and HdhQ150/BL6 mice were anesthetized with 3% isoflurane and transcardially perfused with oxygenated (95% O_2_/5% CO_2_) ice-cold slicing solution containing (in mM) 10 d-glucose, 26 NaHCO_3_, 1.25 NaH_2_PO_4_, 3 KCl, 7 MgCl_2_, 212 sucrose and 0.02 CaCl_2_ (pH 7.2; 306 mOsm) using a tissue slicer (VT1200S; Leica, Wetzlar, Germany). Brains were quickly removed and placed into ice-cold solution in the holding chamber of a tissue slicer (Leica, Wetzlar, Germany), and 250 µm thick coronal acute brain slices were cut in oxygenated ice-cold artificial cerebrospinal fluid (ACSF) containing (in mM): 125 NaCl, 25 d-glucose, 25 NaHCO_3_, 1.25 NaH_2_PO_4_, 2.5 KCl, 2 CaCl_2_, 1 MgCl_2_, pH 7.2 and 306 mOsm. Slices were collected in a holding chamber containing oxygenated ACSF at 37 °C for 15 min and subsequently at room temperature for 45 min before recordings.

During recording, slices were constantly perfused with oxygenated ACSF. Neurons were visualized with an Olympus BX51WI upright microscope (Olympus, Shinjuku, Japan) fitted with a 4× objective (Plan N, NA 0.1; Olympus, Shinjuku, Japan), a 40× water-immersion objective (LUMPlan FI/IR, NA 0.8w; Olympus, Shinjuku, Japan) and a CCD camera (XM10, Olympus, Shinjuku, Japan).

For electrophysiological patch-clamp experiments, recording and stimulation electrodes were controlled by a SM7 remote unit and control box (Luigs and Neumann, Ratingen, Germany). Glass capillaries (Hilgenberg, Malsfeld, Germany) were filled with the following solution for recording of active and passive membrane properties (in mM): 130 K-gluconate, 10 HEPES, 10 phosphocreatine-Na, 10 Na-gluconate, 0.3 GTP, 4 MgATP and 4 NaCl (pH 7.2, adjusted with KOH). For nucleated patches, glass capillaries were filled with the following solution (in mM): 105 K-gluconate, 30 KCl, 10 HEPES, 10 phosphocreatine-Na, 0.3 GTP and 4 MgATP (pH 7.3, adjusted with KOH). For all other whole-cell recordings, the following solution was filled in the glass capillaries (in mM): 120 Cs-gluconate, 10 CsCl, 8 NaCl, 10 HEPES, 10 phosphocreatine-Na, 0.3 GTP, 2 MgATP and 0.2 EGTA (pH 7.3, adjusted with NaOH), and 0.1–0.5% biocytin was additionally added.

Electrical signals were acquired at 10 kHz for miniature excitatory postsynaptic currents (mEPSC) recordings and 50 kHz for all other recordings using an EPC10 amplifier (HEKA, Reutlingen, Germany) and Patchmaster software (HEKA, Reutlingen, Germany). Liquid junction potential was not adjusted. mEPSCs were recorded at a holding potential of −70 mV in ACSF in the presence of 1 µM TTX (Biotrend, Cologne, Germany), hydrobromide (SR95531; Biotrend, Cologne, Germany) and 50 µM APV (Biotrend, Cologne, Germany). Test pulses were delivered every 10 s to monitor series resistance. Active and passive membrane properties were analyzed by applying hyper- and depolarizing current injections.

Synaptic NMDAR- and AMPAR-mediated currents were evoked using a stimulation electrode (chlorinated silver wire inside a borosilicate glass capillary filled with ACSF), which was located at a distance of approximately 100 µm from the patched MSNs. AMPAR- and NMDAR-mediated currents were recorded at holding potentials of −70 and +40 mV, respectively, in ACSF in the presence of hydrobromide (SR95531; Biotrend, Cologne, Germany).

Extrasynaptic NMDAR-mediated currents were analyzed as described previously [97]. Briefly, short pulses (1 m) of 1 mM glutamate were delivered onto nucleated patches of MSNs with a piezo-controlled theta-glass (Hilgenberg, Malsfeld, Germany) filled with a HEPES solution containing (in mM) 135 NaCl, 10 HEPES, 5.4 KCl, 1.8 CaCl_2_, 5 glucose, 0.01 CNQX and 0.01 glycine (pH 7.2), as done previously [98]. The piezo actor and control software (Physik Instrumente; Karlsruhe, Germany) were triggered with the Patchmaster software.

NMDAR-mediated current kinetics were obtained from double exponential fits of current deactivation or decay using the formula: I = A1(e − t/τ1) + A2(e − t/τ2), where A1 and A2 are the amplitudes of the time constants τ1 and τ2, respectively. The weighted deactivation and decay time constant was calculated as: wτ = τ1(A1/(A1 + A2)) + τ2(A2/(A1 + A2)).

### 5.3. Spine and Sholl Analysis

Analysis of neuron anatomy was done as previously described [99]. Briefly, after filling MSNs with a biocytin-containing intercellular solution during electrophysiological analyses, brain slices were removed from the recording chamber, fixed with 4% Histofix (Roth, Germany) and subsequently stained with Streptavidin-Alexa Fluor 594 (dilution of 1:1000; ThermoFisher Scientific Inc., Waltham, MA, USA). Slices were mounted onto slides with ProLong Gold antifade reagent (ThermoFisher Scientific Inc., Waltham, MA, USA).

Proximal and distal dendrites of MSNs in the striatum were imaged with a 63× objective (Olympus, Shinjuku, Japan) on a confocal microscope (Leica SP5 LSM; Leica, Wetzlar, Germany). Subsequent blind deconvolution was performed with Amira software (ThermoFisher Scientific Inc., Waltham, MA, USA). Whole-cell images for Sholl analysis were taken with a 40× objective (Olympus, Shinjuku, Japan).

Semi-automatic spine counting of proximal and distal dendrites and tracing of dendrites of whole cells was carried out with NeuronStudio (version 0.9.92; Computational Neurobiology and Imaging Center Mount Sinai School of Medicine, New York, NY, USA). Sholl analyses of the reconstructed neurons were performed with Neurolucida (version 3.70.2; MBF Bioscience; Williston, VT, USA), with starting radius and radius increment parameter set to 10 µm.

### 5.4. mHTT Immunostaining

Mice were deeply anesthetized with isoflurane and transcardially perfused with phosphate buffered saline (PBS) and 4% PFA (Roth, Karlsruhe, Germany). Then, the brain was extracted and cut into coronal mouse brain slices (70 µm thick) with a tissue slicer (VT1000; Leica, Wetzlar, Germany) containing the striatum were fixed in 4% Histofix (Roth, Germany) and stained with an antibody detecting exclusively huntingtin aggregates as inclusions (monoclonal MW8, dilution 1:10 in 3% BSA, 2% NGS, 0.2% Triton-X-100, PBS buffer corresponding to 5 µg/mL; Developmental Studies Hybridoma Bank, Iowa City, IA, USA). Subsequently, after several washing steps with PBS, the secondary antibody Rabbit anti-Mouse IgG (H + L) Alexa Fluor 594 was added (A27027, dilution of 1:1000, ThermoFisher Scientific Inc., Waltham, MA, USA). Nuclei were visualized using a DAPI staining (D1306, dilution of 1:10,000; ThermoFisher Scientific Inc., Waltham, MA, USA). After several washing steps with PBS, slices were mounted on a glass slide with ProLong Gold antifade reagent (life technologies).

Slices were imaged with a 40× objective (for whole-cell images) or a 63× objective (for dendrite close ups) objective (Leica, Wetzlar, Germany) on a confocal microscope (Leica SP5 LSM; Leica, Wetzlar, Germany).

### 5.5. Behavior Experiments

Mice were tested in a battery of behavior experiments: on day 1, mice were tested in the Open Field test and underwent a Rotarod training session. On day 2, mice underwent a second Rotarod training. On day 3, mice performed the Rotarod and Catwalk experiment.

#### 5.5.1. Open Field

Exploratory behavior and activity of mice was investigated with a standard Open Field test. The box measured 42 × 42 cm^2^ and was subdivided into 16 squares and a squared center area (see Figure 6a). Activity was tracked and analyzed with the SYGNIS tracker software (version 4.14; Sygnis Bioscience, Heidelberg, Germany).

All animals were placed in the same corner and tracked for 5 min. Time as well as the distance travelled in each square was listed. Several parameters were calculated: the total travelled distance, the travelled distance in the periphery (light grey in Figure 6a) versus the travelled distance in the center of the field (dark grey in Figure 6a), the time spent near the wall versus the time spent in the center of the box and the visits spent in the different areas.

#### 5.5.2. Rotarod

Mice were trained three times a day on the accelerating Rotarod (Ugi Baslie; Varese, Italy) on two consecutive days. The training was performed under the same conditions as the test trials. The Rotarod was set to accelerate starting from four until 40 rounds per minute within 8 min. Latency to fall off the Rotarod was measured. Mice that did not fall off within 8 min were removed from the Rotarod by hand and their latency to fall was set to 8 min. Each mouse was tested three times with a resting time of 20 min in between the experiments, and the mean of the three time points was calculated.

#### 5.5.3. Catwalk/Gait Analysis

Gait analysis was performed on a Catwalk as described previously [100]. The Catwalk system consisted of an enclosed corridor (4.5 cm broad) with a glass walkway. Fluorescent light is internally reflected within the walkway. Scattering of the light when the mouse paws contact the glass floor produces paw prints. Sessions were recorded and analyzed with the CatWalk XT 10.6 software (Noldus, Wageningen, The Netherlands). For each mouse, three runs with a maximal run duration of 10 s were recorded and runs with rather constant speed were analyzed (speed variance < 100%). The mean of the three time points was calculated for the final gait values. Footprints were classified automatically and adjusted manually, if necessary.

Gait parameters were analyzed as done previously [100]. The swing speed describes the speed of a paw during swing. The swing phase time (swing) describes the time one paw is in the air during one step. Support from the abdomen is observed when not only the paws are specified on the glass plate but also the abdomen which can relieve wait from the hint paws [19]. The print of the abdomen was classified automatically as the prints of the paws and was manually adjusted, if necessary. It describes the total duration of abdominal contact with the glass plate (given in %) over the entire run of the animal.

### 5.6. Statistical Analysis

Statistical analyses were performed in GraphPad Prism Version 6.07 (GraphPad Software; San Diego, CA, USA). Data were tested for normal distribution with D’Agostino-Pearson omnibus normality test. Normally distributed data were analyzed by *t*-test, and not-normally distributed data were analyzed by a Mann–Whitney-test. Bar graphs show the median ± interquartile range (IQR) for normally and not-normally distributed data. A *p*-value < 0.05 was considered statistically significant (* = *p* < 0.05, ** = *p* < 0.01, *** = *p* < 0.001). Single data points are depicted as grey circles in bar graphs.

## Supplementary Materials

The following are available online at https://www.mdpi.com/article/10.3390/ijms222312664/s1.
