# Supplementary material for: C57BL/6 Background Attenuates mHTT Toxicity in the Striatum of YAC128 Mice
Source: Int J Mol Sci. 2021 Nov 23;22(23):12664. doi: 10.3390/ijms222312664 (PMC8657915; doi:10.3390/ijms222312664)
Supplement: Supplementary file 1 [file ijms-22-12664-s001.zip › S1.pdf]

Supplemental Figure S1: Functional synapses are not impaired in 3-month-old and 18-month-old YAC128/BL6 mice

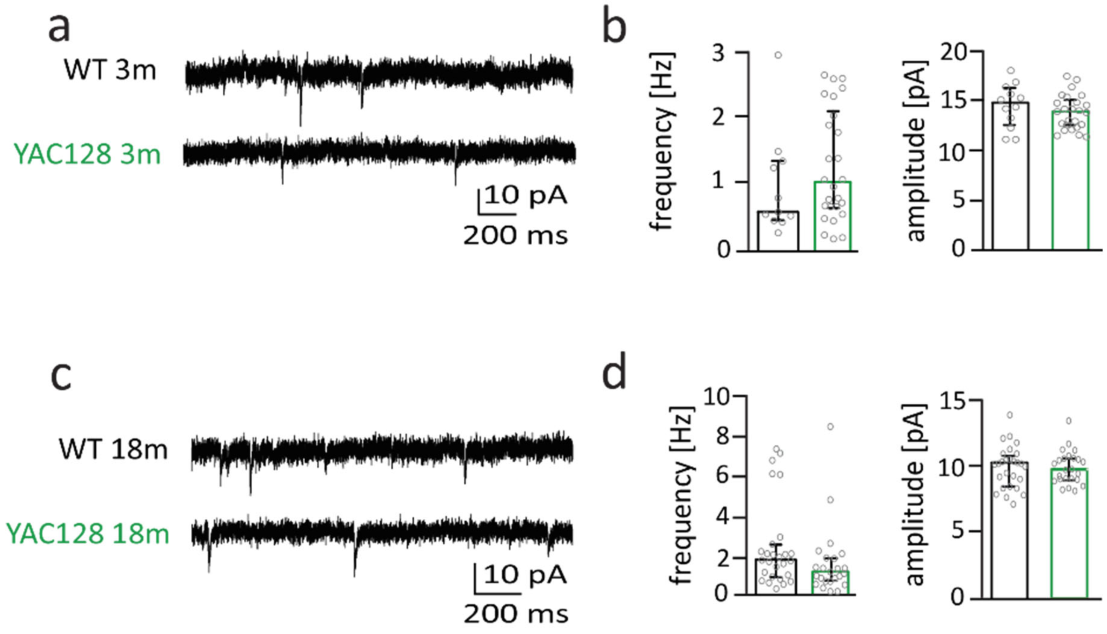

(a) Sample traces of mEPSC recordings from MSNs of 3-month-old WT and YAC128/BL6 mice. (b) Bar graphs of mEPSC frequency and peak amplitudes of MSNs from 3-month-old WT (n= 12) and YAC128/BL6 (n=25) mice. (c) Sample traces of mEPSC recordings from MSNs of 18-month old WT and YAC128/BL6 mice. (d) Bar graphs of mEPSC frequency and peak amplitudes of MSNs from 18-month-old WT (n= 27) and YAC128/BL6 (n=24) mice. Bar graphs depict median ± IQR.
