# Supplementary material for: C57BL/6 Background Attenuates mHTT Toxicity in the Striatum of YAC128 Mice
Source: Int J Mol Sci. 2021 Nov 23;22(23):12664. doi: 10.3390/ijms222312664 (PMC8657915; doi:10.3390/ijms222312664)
Supplement: Supplementary file 1 [file ijms-22-12664-s001.zip › S2.pdf]

Supplemental Figure S2: Behavior is impaired in 9-month-old female and male YAC128/BL6 mice

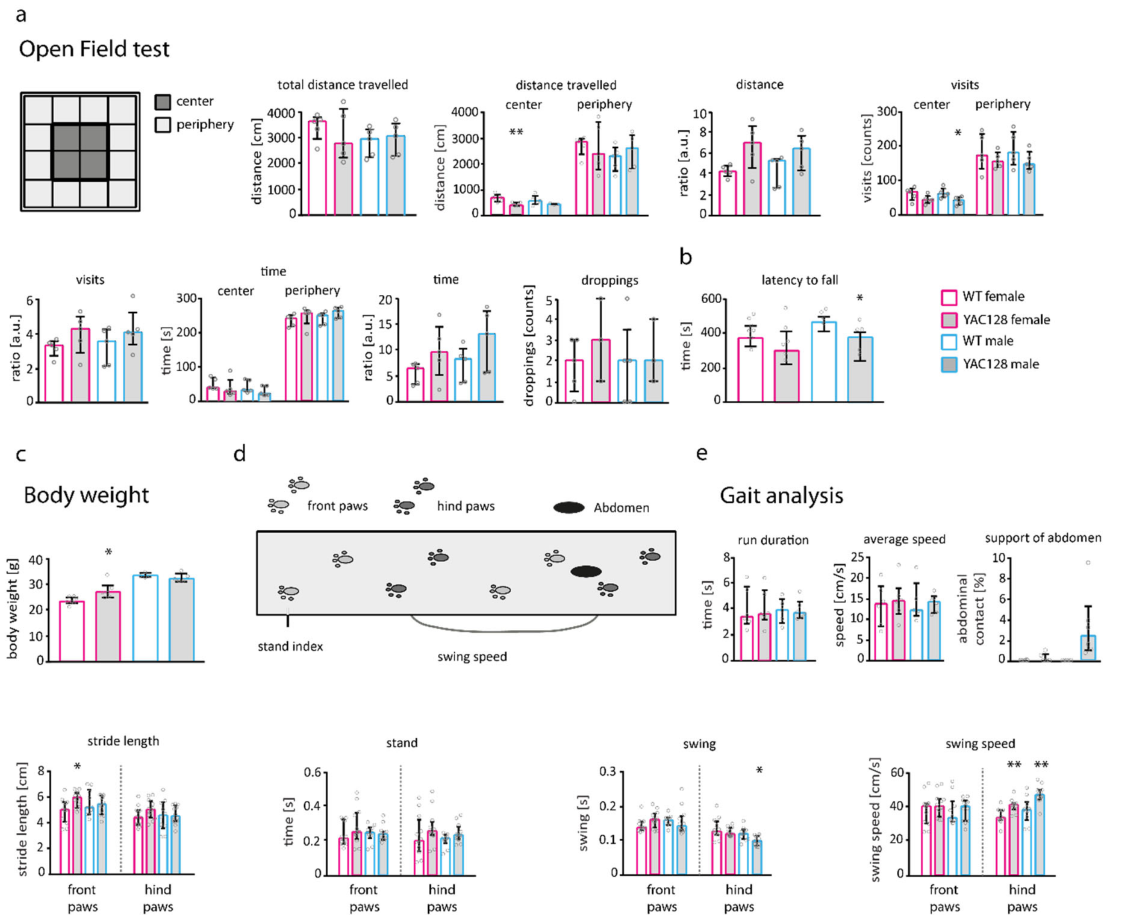

Data of Figure 6 separated by sex. (a—left) Scheme of the Open Field divisions with center and periphery. (a—right) Bar graphs of Open Field behavioral parameters of WT (female n = 5; male = 5) and YAC128/BL6 (female n = 5; male n = 6) mice. (b) Bar graph of the latency to fall off the Rotarod for male and female WT (n females = 7; males = 6) and YAC128/BL6 (n females = 4, males = 5) mice. (c) Bar graph of the median body weight of female and male of WT and YAC128/BL6 mice. (d) Scheme of the gait analysis (Catwalk) setup. (e) Bar graphs of gait analysis parameters from WT (female n = 5; male = 5) and YAC128/BL6 (female n = 5; male n = 6) mice. Bar graphs are depicted as median ± IQR. * = $p < 0.05$, ** = $p < 0.01$
